# Sample Augmentation Using Enhanced Auxiliary Classifier Generative Adversarial Network by Transformer for Railway Freight Train Wheelset Bearing Fault Diagnosis

**DOI:** 10.3390/e26121113

**Published:** 2024-12-20

**Authors:** Jing Zhao, Junfeng Li, Zonghao Yuan, Tianming Mu, Zengqiang Ma, Suyan Liu

**Affiliations:** 1 School of Traffic and Transportation, Shijiazhuang Tiedao University, Shijiazhuang 050043, China; rxzhaojing@126.com; 2 Hebei Province University Road Traffic Perception and Intelligent Application Technology Research and Development Center, Hebei Jiaotong Vocational and Technical College, Shijiazhuang 050011, China; 3 School of Computer Science, South China Business College Guangdong University of Foreign Studies, Guangzhou 510545, China; 201093@gwng.edu.cn; 4 College of Career Technology, Hebei Normal University, Shijiazhuang 050043, China; yuanzonghao2006@163.com; 5 State Grid Shijiazhuang Electric Power Supply Company, Shijiazhuang 050021, China; caleb_good@126.com; 6 School of Electrical and Electronic Engineering, Shijiazhuang Tiedao University, Shijiazhuang 050043, China; liusuyan@stdu.edu.cn; 7 Hebei Provincial Collaborative Innovation Center of Transportation Power Grid Intelligent Integration Technology and Equipment, Shijiazhuang Tiedao University, Shijiazhuang 050043, China

**Keywords:** fault diagnosis, generative adversarial network, transformer, wheelset bearing, cross-entropy

## Abstract

Diagnosing faults in wheelset bearings is critical for train safety. The main challenge is that only a limited amount of fault sample data can be obtained during high-speed train operations. This scarcity of samples impacts the training and accuracy of deep learning models for wheelset bearing fault diagnosis. Studies show that the Auxiliary Classifier Generative Adversarial Network (ACGAN) demonstrates promising performance in addressing this issue. However, existing ACGAN models have drawbacks such as complexity, high computational expenses, mode collapse, and vanishing gradients. Aiming to address these issues, this paper presents the Transformer and Auxiliary Classifier Generative Adversarial Network (TACGAN), which increases the diversity, complexity and entropy of generated samples, and maximizes the entropy of the generated samples. The transformer network replaces traditional convolutional neural networks (CNNs), avoiding iterative and convolutional structures, thereby reducing computational expenses. Moreover, an independent classifier is integrated to prevent the coupling problem, where the discriminator is simultaneously identified and classified in the ACGAN. Finally, the Wasserstein distance is employed in the loss function to mitigate mode collapse and vanishing gradients. Experimental results using the train wheelset bearing datasets demonstrate the accuracy and effectiveness of the TACGAN.

## 1. Introduction

As a crucial part of train bogies, wheelset bearings are vital for load transmission and motion conversion [1]. These bearings endure various alternating loads during train operations. Once a component’s surface sustains local damages, the fault can progressively worsen under dynamic loads, leading to a decline in the functionality of the entire transmission system and potentially causing significant accidents [2,3]. Thus, online detection and fault diagnosis of wheelset bearings are essential.

Traditional fault diagnosis methods primarily rely on signal processing to analyze bearing faults by denoising and extracting fault features from the vibration signals of wheelset bearings [4,5]. By comparing frequencies derived from the dimensions of bearing components in different damaged areas, the health status and location of damaged parts are determined. In this context, Cao H et al. [6] utilized the empirical wavelet transform (EWT) for vibration signal analysis in fault diagnosis. Moreover, Li H et al. [7] introduced an improved ensemble empirical mode decomposition (EEMD) time–frequency analysis method based on the Improved Adaptive Resonance Technique (IART), enhancing the denoising capability of fault-related pulse signals and effectively detecting rolling bearing faults. Zheng J et al. [8] introduced a rolling bearing fault diagnosis method based on composite multiscale fuzzy entropy (CMFE) and ensemble support vector machines (ESVMs), which can effectively classify fault categories and severity levels of rolling bearings. Yu W et al. [9] introduced a probabilistic ensemble learning-based Bayesian network (PEL-BN) strategy for online fault diagnosis technology. Jiao J et al. [10] introduced a favorable Sparse Local FDA (SLFDA) model, which utilizes the local data structure characteristics from both sample and variable dimensions, and significantly enhances fault performance and model explainability.

In the era of big data, deep learning exhibits unique feature extraction capabilities. Deep learning can automatically extract useful features from samples, perform end-to-end fault classification, and avoid dependence on signal-processing techniques and prior knowledge. Techniques such as convolutional neural networks (CNNs) [11,12], recurrent neural networks (RNNs) [13,14], generative adversarial networks (GANs) [15], and autoencoders (AEs) [16,17,18] are employed in this context. For instance, Yu W et al. [19] utilized a Broad Convolutional Neural Network (BCNN) with incremental learning capability, which can better capture the characteristics of the fault process and effectively update diagnostic model. N. Daldal et al. [20] utilized two CNNs with different kernel sizes for automatic signal feature extraction and employed long-term and short-term memory networks to determine fault types. Furthermore, Yang et al. [21] introduced the CGAN-2-D-CNN fusion diagnostic method for diagnosing small bearing faults.

Despite the promising results achieved by wheel bearing fault classification algorithms, several challenges persist. First, these methods require labeled samples, and it is often impractical to label wheel-bearing fault samples during collection [22,23]. Second, the methods need a sufficient and balanced number of trained samples; otherwise, the diagnostic accuracy may decrease or overfitting may occur [24]. To address the issue of unbalanced samples in bearing fault diagnosis, data augmentation methods (DAMs) like rotation, translation, scaling, flipping, and cropping are often employed to expand data samples. Li W et al. [25,26] proposed an enhanced GAN model (MACGAN) with a new framework design, incorporating a classification function to develop a new ACGAN framework. The ACGAN structure includes a CNN, providing discriminative and classification capabilities [27]. Another approach is to input noise and category labels into the generator, enabling the discriminator to identify fault types while assessing sample authenticity. Studies show that the ACGAN exhibits a promising performance for data augmentation. For instance, Zou L et al. [28] applied the ACGAN in the maritime field to enhance the resolution of sliced ground images. Moreover, Jin et al. [29] proposed a multi-layer branch ACGAN for signal expansion. However, the ACGAN faces several problems: (1) Generators in an ACGAN typically use CNNs with hierarchical structures composed of multiple convolutional and pooling layers, resulting in a cumbersome data generation process and long training times. (2) Most ACGAN methods employ Jensen Shannon (JS) divergence for their loss functions, leading to instability and vanishing gradients due to its discrete nature. (3) The ACGAN discriminator is tasked with discrimination and classification simultaneously [30,31]. In summary, addressing the limitations of the traditional ACGAN, achieving efficient and high-quality sample generation, and accurately diagnosing wheelset-bearing faults are of significant importance.

As a network structure built on a self-attention mechanism, the transformer has been extensively utilized in the field of Natural Language Processing (NLP) due to its powerful scaling capabilities and ability to learn long-range dependencies [32,33,34,35]. Recently, researchers have increasingly applied the transformer’s robust modeling capabilities to computer vision (CV) and bearing fault diagnosis [36,37,38,39]. Based on the literature survey performed, this paper proposes the transformer and auxiliary classifier generative adversarial network (TACGAN) to address issues in data augmentation and fault classification under various working conditions, aiming for balanced fault categories and high-precision fault classification. The TACGAN generates vibration signals for different fault categories of bearings and includes a transformer generator, a transformer discriminator, and a CNN classifier. The performance of the TACGAN was evaluated using collected vibration data, demonstrating its effectiveness in generating high-quality fault samples across different categories, surpassing existing ACGAN methods. The main contributions of this article can be summarized as follows:

(1) To eliminate the cumbersome loop structure of convolutional layers; transformer networks replace CNNs in the generator and discriminator.
(2) To avoid vanishing gradients, exploding gradients, and overfitting issues, the Wasserstein distance is introduced into the new cross-entropy loss function, ensuring stability.
(3) To prevent the overlap of discrimination and classification tasks in the ACGAN discriminator, the classifier is separated from the discriminator as an independent component.

This article is organized as follows: Section 2 explains the basic principles of the ACGAN and transformers. Section 3 introduces the proposed TACGAN model and its training process. Section 4 presents experimental validations demonstrating the effectiveness of sample generation and fault classification. Finally, the main achievements are summarized in Section 5.

## 2. Basic Theory

### 2.1. Auxiliary Classifier Generative Adversarial Networks

**Figure 1:**
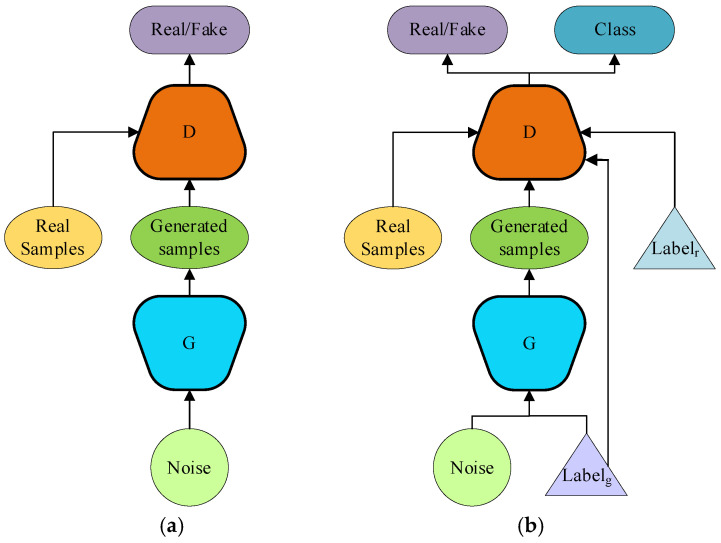
Structures of GAN (**a**) and ACGAN (**b**).

The architecture of the ACGAN closely resembles that of a standard GAN, with the key distinction being its use of label information as a conditional probability for input samples. Unlike the GAN, the ACGAN incorporates a more sophisticated design by effectively utilizing the discriminator. This discriminator not only distinguishes between real and fake samples but also categorizes them. By evaluating the categories of the generated samples, the discriminator can more effectively transmit the loss function LS and classification loss LC to the generator. This dual-feedback mechanism enables the generator to more accurately learn the true distribution of samples corresponding to each label [40,41,42]. Figure 1 illustrates the similarities and differences between the ACGAN and the Conditional GAN (CGAN).

(1) $$L_{S}=E_{r}\left[logP(s=s_{r}|x)\right]+E_{f}\left[logP(s=s_{f}|G\left(a,c_{f}\right))\right]$$

(2) $$L_{C}=E_{r}\left[-logP(c=c_{r}|x)\right]+E_{f}\left[-logP(c=c_{f}|G\left(a,c_{f}\right))\right]$$

where *L_S_* denotes the loss function used to determine the authenticity of the samples, while *L_C_* represents the loss function used to classify the type of data. Moreover, *E_r_* and *E_f_* are the expected values of *x* and *z*, which follow the distribution of real samples and noise, respectively. *G*(*a*, *c_f_*) denotes the generated samples, where a represents the noise input. *c_r_* denotes the label for the real sample *x*, and *c_f_* represents the label for the sample *G*(*a*, *c_f_*) generated by the generator.

### 2.2. Transformer Encoder

The transformer, primarily used for processing sequential data like text, was introduced by a Google research team in 2017, with a focus on machine translation scenarios [43]. Compared to previously used RNNs and CNNs, the transformer offers faster processing, requires fewer parameters, mitigates the challenge of long-distance information retention, allows for parallel feature extraction, and incorporates global feature modeling to reduce the learning cycle [44]. Consequently, the transformer model has been widely adopted and has demonstrated outstanding performance across various industries.

**Figure 2:**
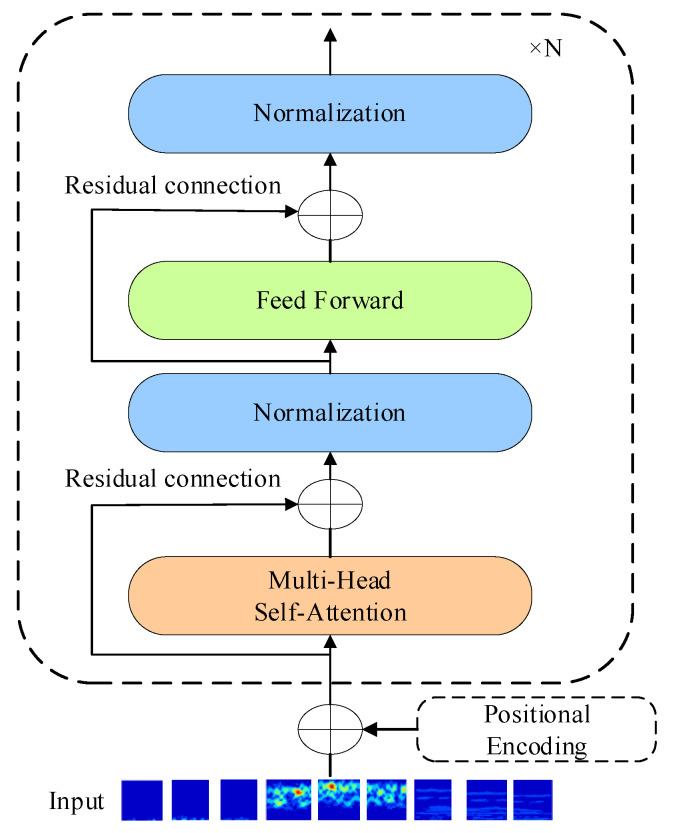
Structure of a transformer encoder network.

A typical transformer architecture consists of an encoder and a decoder, with the encoder exhibiting a robust feature extraction capability. For wheelset bearing classification, the encoder is utilized, as depicted in Figure 2. The transformer encoder is composed of N identical encoding units, each layer comprising two primary components: a multi-head self-attention (MSA) module and a feedforward network (FFN) module. Residual connections are employed between these two layers, and each layer is followed by a layer normalization (LayerNorm), structured as follows:

(3) $$X_{MSA}=LayerNorm\left(MSA\left(X_{in}\right)+X_{in}\right)$$

(4) $$X_{out}=LayerNorm\left(MLP\left(X_{MSA}+X_{MSA}\right)\right)$$

**Figure 3:**
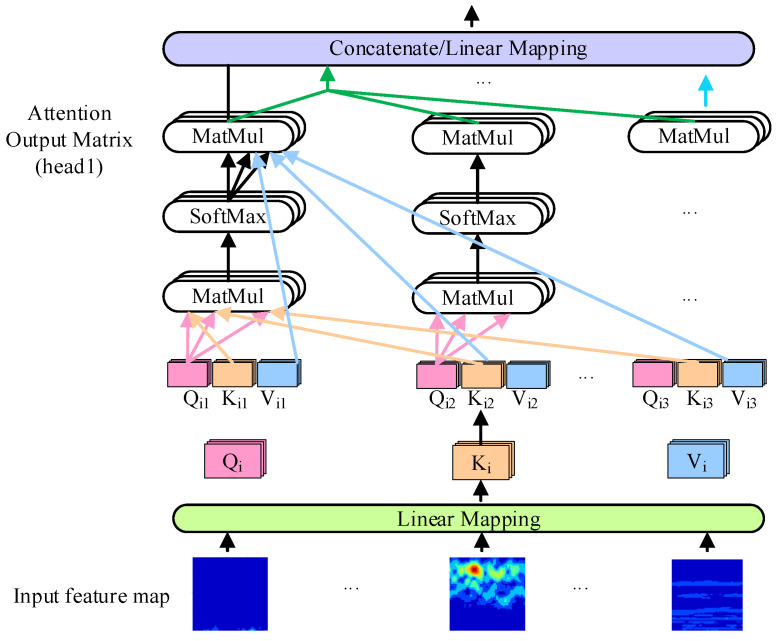
Structure diagram of the multi-head self-attention.

Firstly, through the parameter matrix, the MSA mechanism maps the input two-dimensional embedded image *x*, resulting in three matrices: the query *Q_i_*, the key *K_i_*, and the value *V_i_*. In this formulation, *i* ranges from 1 to *H*, where *H* represents the total number of heads. The weighted sum is then obtained by applying scaled dot product attention on *V* and computing the weight matrix by calculating *Q* and *K*. This process is crucial for enhancing the ability to learn global features via MSA, which is essential for the effective diagnosis of the transformer [45]. The MSA function of the *i*-th head is defined as shown in Figure 3.

(5) $$Attention^{headi}\left(Q_{i},K_{i},V_{i}\right)=softmax(\frac{Q_{i}K_{i}^{T}}{\sqrt{d_{k}}})V_{i}$$

where *d_k_* denotes the dimension of *Q* and *K*, and $1/\sqrt{d_{k}}$ is a parameter introduced to balance data and model convergence. Moreover, *Q_i_*, *K_i_*, and *V_i_* are defined as shown below:

(6) $$Q_{i}=XW_{i}^{Q},K_{i}=XW_{i}^{K},V_{i}=XW_{i}^{V}$$

where the parameter matrices $W_{i}^{Q}$, $W_{i}^{K}\in R^{d\times d_{k}}$, $W_{i}^{V}\in R^{d\times d_{v}}$, and *d_v_* represents the dimension of value.

MSA gathers h individual attention sets. Each set of transformed queries, keys, and values is processed in parallel through attention aggregation. These h attention-focused outputs are then concatenated and transformed through another learnable linear projection to produce the final output. This process can be mathematically expressed as follows:

(7) $$MultiHead\left(Q,K,V\right)=Concat\left(Attention^{head1},Attention^{head2},\ldots ,Attention^{headh}\right)W^{O}$$

where $W^{O}\in R^{h.d\times d}$ represents the linear projection after concatenation, and $d_{k}=d_{v}=d/h$.

Then, the FFN module, structured as an MLP, includes a nonlinear transformation with an activation function followed by a linear transformation. In this module, the MLP first uses a nonlinear layer to perform the nonlinear dimensionality raising operation on the input and then uses the linear layer to carry out linear dimensionality reduction operation to extract features. This process can be mathematically described as follows:

(8) $$MLP\left(X_{MSA}\right)=Activation\left(X_{MSA}W_{MLP\_1}+b_{1}\right)W_{MLP\_2}+b_{2}$$

where $W_{MLP\_1}\in R^{d\times d_{MLP}}$ and $W_{MLP\_2}\in R^{d\times d_{MLP}}$ are the weights, $b_{1}\in R^{d_{MLP}}$ and $b_{2}\in R^{d}$, and (·) represents the activation fuction. dMLP denotes the embedding dimension of the nonlinear transformations, *d_MLP_* > *d*.

To capture the relative positions between input data, a position information vector is added to the initial vector before the MSA mechanism. Finally, the token embedding and position encoding are added together. The position encoding is as follows:

(9) $$PE\left(pos,i\right)=\left\{\begin{array}{c} sin(w_{k}\cdot pos),i=2k\\ cos(w_{k}\cdot pos),i=2k+1 \end{array}\right.$$

(10) $$w_{k}=\frac{1}{1000^{2k/d}},k=1,2,\ldots ,d/2$$

## 3. Proposed Framework

**Figure 4:**
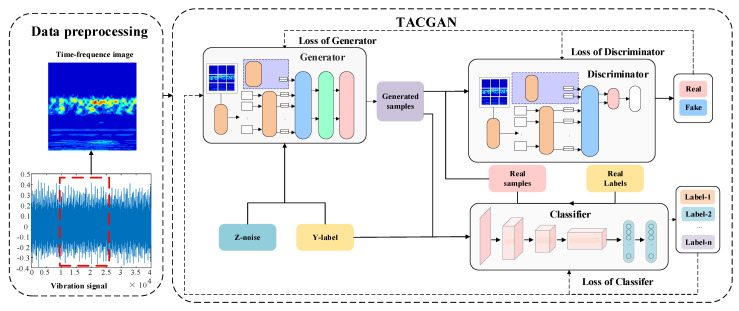
The framework of TACGAN.

To address the challenges of combined identification and classification within the discriminator, this study proposes separating these functions by introducing an independent classifier. This section outlines the comprehensive architecture of the TACGAN. It provides a detailed explanation of the processes involved in sample generation, recognition, classification, and the formulation of a loss function designed for balanced sample generation. The schematic structure of the TACGAN is depicted in Figure 4.

### 3.1. TACGAN Generator

**Figure 5:**
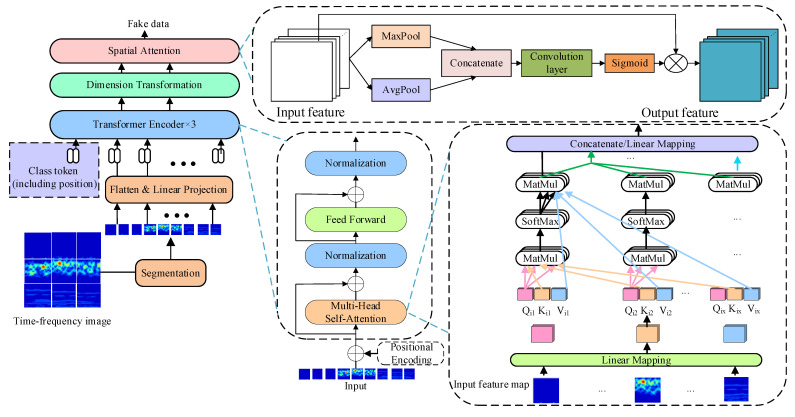
The architecture of generator transformer network.

The TACGAN generates high-quality time–frequency diagrams of wheelset bearings by extracting deep global features from 2D time–frequency images, which are transformed from 1D vibration signals using continuous wavelet transform [46]. It is worth noting that the generated time–frequency images closely resemble real ones. Traditional GAN generators that use CNN networks suffer from computational inefficiencies due to tedious iterations with increasing depth. To address this, the CNN network in the traditional GAN is replaced with a transformer encoder in this paper. The transformer encoder, which has a simpler structure, enables parallel computing and saves GPU space. The generator structure of the TACGAN is shown in Figure 5.

In the TACGAN, the transformer’s input is a 2D time–frequency image, which is divided into tiled 2D image patches, where $x\in R^{H\times W\times C}$, and $x_{p}^{i}\in R^{z\times \left(P1.P2.C\right)}$. The resolution of the input image is specified by H and W, and C represents the number of channels. The resolution of each patch is determined by *P*_1_ and *P*_2_, and *Z* denotes the number of patches. The index i of $x_{p}^{i}$ ranges from 1 to *Z*, with *Z =* (*HW*)/(*P*_1_*P*_2_). A token representing the fault label is added at the beginning of the sequence during random initialization. This token can transfer information by interacting with other vibration signal tokens and positional information during the training process, facilitating learning class information from the input image. With the addition of the fault label token, the image patch sequence length becomes *Z +* 1. Positional encoding is used to extract the positional information of the images. Thus, the transformer’s input can be represented as follows:

(11) $$X_{in}=\left[x_{class};x_{p}^{1}W^{P};x_{p}^{2}W^{P};\ldots ;x_{p}^{Z}W^{P}\right]+E_{pos}$$

where $W^{P}\in R^{\left(P1.P2.C\right)\times d}$ represents the linear projection. The architecture of the transformer generator is shown in Figure 5. The input consists of the fault tag token and the 2D time–frequency image, which is divided into *Z* patches. These inputs are then fed into the transformer encoder module 1.

### 3.2. TACGAN Discriminator

**Figure 6:**
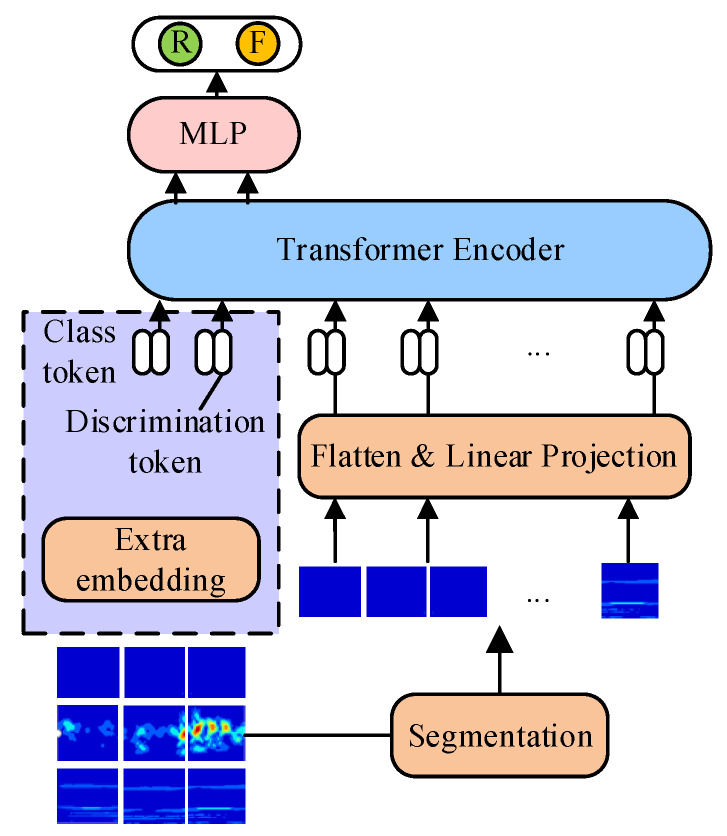
The architecture of discriminator transformer network.

When training convolutional-based neural networks, performing fault diagnosis and authenticity discrimination within the same convolutional network can lead to mutual interference, reducing the network’s accuracy and classification capability. To address this issue, an independent classifier is established, allowing the discriminator to focus solely on sample authenticity analysis. In the TACGAN, the discriminator processes real samples, generates samples, and labels their authenticity. The internal structure of the transformer encoder in the discriminator is similar to that of the generator. An authenticity tag token is added to the header of the transformer encoder, which then exchanges information with the batch of 2D time–frequency images to determine the probability that the output is genuine, as illustrated in Figure 6. The input to the discriminator (*D*) includes a 2D time–frequency image and an authenticity tag token, resulting in an input sequence length of *Z* + 1. The input format for the discriminator is represented as follows:

(12) $$X_{D\_in}=\left[x_{fr};x_{p}^{1}W^{P};x_{p}^{2}W^{P};\ldots ;x_{p}^{Z}W^{P}\right]+E_{pos}$$

The concrete parameters of the generator and discriminator for the TACGAN proposed are shown in Table 1.

### 3.3. TACGAN Classifier

The input of the TACGAN independent classifier is the generated real samples and fault tags, as shown in Figure 4, and the classifier is composed of a CNN-based model. The detailed parameters are listed in Table 2.

### 3.4. Loss Function of TACGAN

In the TACGAN, Wasserstein distance is used to the losses. Wasserstein distance may provide a meaningful gradient as follows:

(13) $$W\left(P_{r},P_{g}\right)=\underset{\gamma \sim \prod \left(P_{r},P_{g}\right)}{inf}E_{\left(x,y\right)}\left[\left\|x-y\right\|\right]$$

where $\prod \left(P_{r},P_{g}\right)$ is a set of joint distributions combined with *P_r_* and *P_g_*. For each possible joint distribution γ, the real sample x, and generate sample g can be obtained from (*x*, *g*)~γ, and the distance ∥x−g∥ between the two sample pairs is calculated in this joint distribution γ. The loss of the TACGAN is as follows:

(14) $$L_{C}^{R}=E_{x\sim P_{r\left(x\right)}}\left[-logP\left(c=c_{r}\left|x\right.\right)\right]$$

(15) $$L_{C}^{G}=E_{z\sim P_{z\left(z\right)}}\left[-logP\left(c=c_{g}\left|G\left(z,c_{g}\right)\right.\right)\right]$$

(16) $$L_{D}=E_{x\sim P_{r\left(x\right)}}[D\left(x\right)-E_{zP_{z\left(z\right)}}\sim [D\left(G\left(z,c_{g}\right)\right)]]$$

(17) $$L_{G}=-E_{z\sim P_{z\left(z\right)}}\left[D\left(G\left(z,c_{g}\right)\right)\right]+0.5\times L_{C}^{R}+0.5\times L_{C}^{G}$$

(18) $$L_{C}=\lambda_{1}L_{C}^{R}+\lambda_{2}L_{C}^{G}$$

where *L_D_*, *L_G_* and *L_C_* are the loss of D, G, and C. $L_{C}^{R}$ is the loss function of C for real data, and $L_{C}^{G}$ is the loss of C for generated data. $\lambda_{1}$ and $\lambda_{2}$ are the ratio factors of C loss in TACGAN. The TACGAN is needed to train, listed in Algorithm 1.


**Algorithm 1:** The training algorithm of the TACGAN       Wheelset bearing fault diagnosis with the proposed model  the Inputed labeled samples X = {(xj, aj)};1: Initialize model parameters:2: For j = 1 to N do:3:   For i = 1 to 5 do:4:    Extract a batch real datas{x}, Construct the noise with the label information{a, y};5:    Calculate G(a, cf);6:    LD = Ereal[D(x) − Efalse[D(G(a, cf))]];7:   End For8:   Extract a batch real datas{x, y}, Construct the noise with the label information{a, y};9:    Calculate G(a, cf);10:   LrC = Ereal[−logP(c = cr|x)]; LfC = Efalse[−logP(c = cr|x)];11:   LG = Efalse[D(G(a, cf))] + λLfC;12:   θC←Adam(LC);13:   θG←RMSProp(LG);14: End For


## 4. Experimental Verification

### 4.1. Datasets

**Figure 7:**
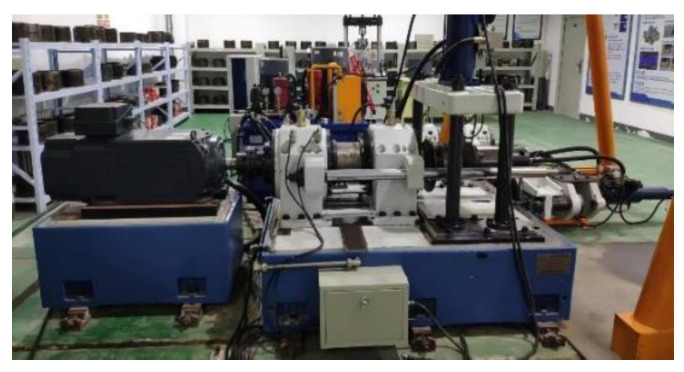
Picture of wheelset bearing experiment platform.

**Figure 8:**
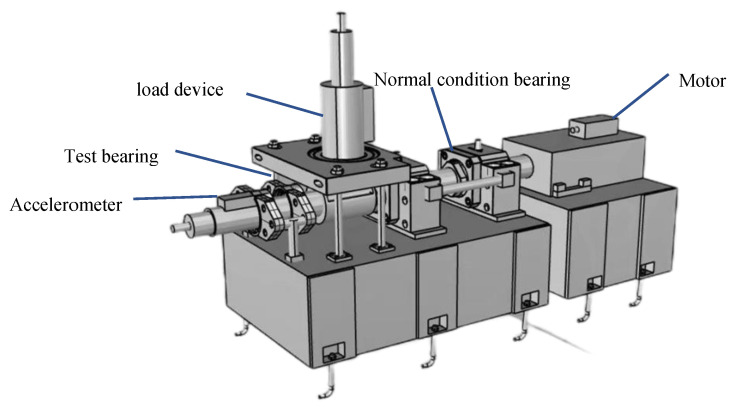
Schematic of experiment platform.

To evaluate the performance of the TACGAN, vibration signal data from wheelset bearings, measured using a high-speed train wheelset bearing comprehensive test platform, are utilized. As shown in Figure 7, the platform includes a driving motor, loading device, bearing, and test bearing. An accelerometer for vibration signal acquisition is mounted on the test wheelset bearing, as depicted in Figure 8. This accelerometer has a sensitivity of 2.505 mV/m/s^2^.

**Figure 9:**
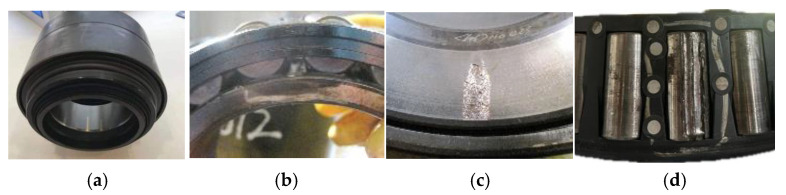
Photo of the test bearing. (**a**) N; (**b**) I; (**c**) O; (**d**) R.

During the test, the sampling frequency is set to 12.8 kHz. The wheelset bearing under test exhibits four states: normal (N), inner ring fault (I), outer ring fault (O), and rolling element fault (R). Photographs of the test wheelset bearing are shown in Figure 9, with detailed specifications provided in Table 3.

**Figure 10:**
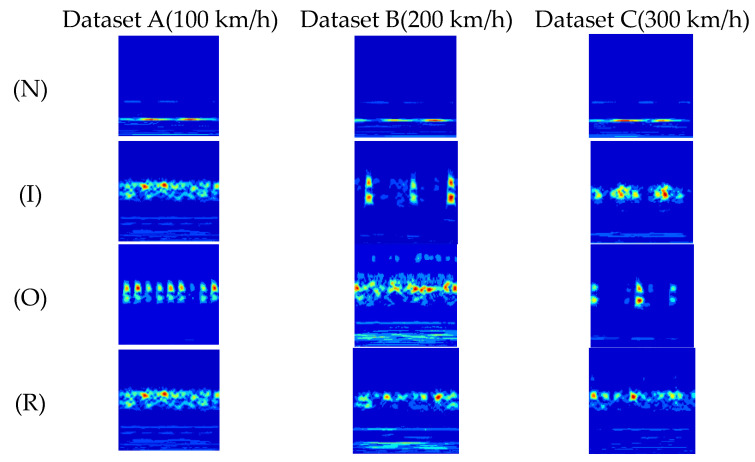
Data of wheelset fault bearings.

Fault samples are collected by the linear velocity of the wheelset bearing: 100 km/h, 200 km/h, and 300 km/h, corresponding to datasets A, B, and C, respectively. The operational load of the wheelset bearing is 5 tons, resulting in 12 experimental datasets for verification. Initially, the original 1D vibration signals are transformed into 2D time–frequency images using the continuous wavelet transform method, which serves as the input for the TACGAN model. The transformation employs the complex Morlet wavelet with a scale of 128, center frequency, and bandwidth of 2. The resulting 256 × 256 time–frequency images of real and generated samples are shown in Figure 10. Each sample comprises 1024 data points, and 2000 samples are used for each experimental dataset, totaling 24,000 samples from the 12 datasets. Detailed information about the train bearing datasets is provided in Table 4. The data are split into training and experimental sets in a 7:3 ratio to test the network’s generalization performance.

Experimental validation is performed using a Python 3.8 script, specifically designed for this purpose. The deep learning architecture is built on PyTorch 1.3, ensuring a robust and efficient framework. Experiments and training processes are conducted on a Windows 10 system, powered by an Intel Xeon Gold 6148 CPU, 16 GB of RAM, and an RTX 2080 Ti GPU with 4 GB of dedicated memory, facilitating high-performance computations and accelerating the training procedures.

### 4.2. Sample Generation and Fault Diagnosis

#### 4.2.1. Performance of TACGAN

**Figure 11:**
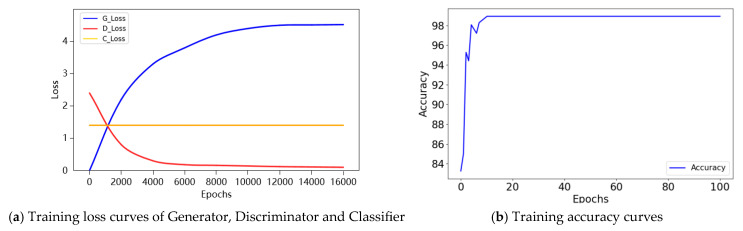
Training process of TACGAN.

To evaluate the performance of the TACGAN under conditions of sample scarcity, experiments were conducted using the aforementioned samples. Figure 11a reveals that after 10,000 epochs of training, the model reached stable accuracy across all three components. Remarkably, Figure 11b demonstrates that the classifier model reached stable accuracy after approximately 20 epochs. This observation demonstrates the training process of the three modules: the generator, discriminator, and classifier. Figure 11 shows that the proposed TACGAN exhibits significant training stability, with all components working in tandem to achieve a rapid stable operational state and maintaining it until the training concludes.

**Figure 12:**
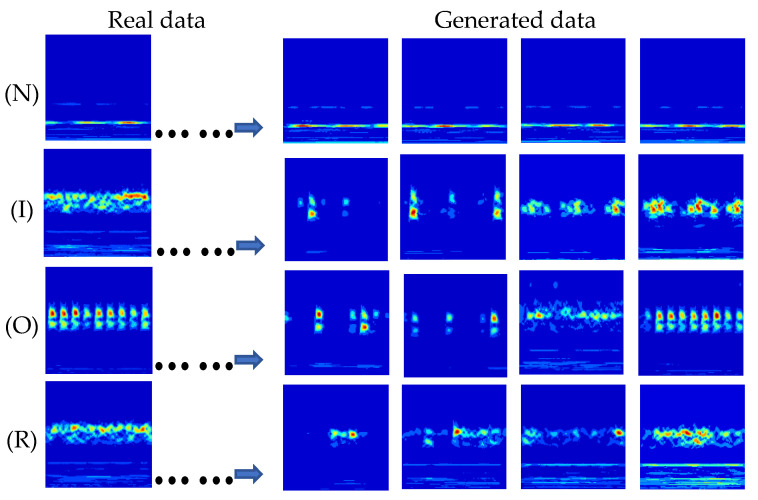
Real data and generated data of TACGAN.

A common issue with many GANs is mode collapse, where the generated samples lack diversity, causing the discriminator to be deceived by repetitive patterns. Mode collapse can be identified through visual inspection. As shown in Figure 12, the generated samples display a high level of diversity and closely resemble real samples in the temporal domain. Notably, there is no evidence of mode collapse in the generated samples.

**Figure 13:**
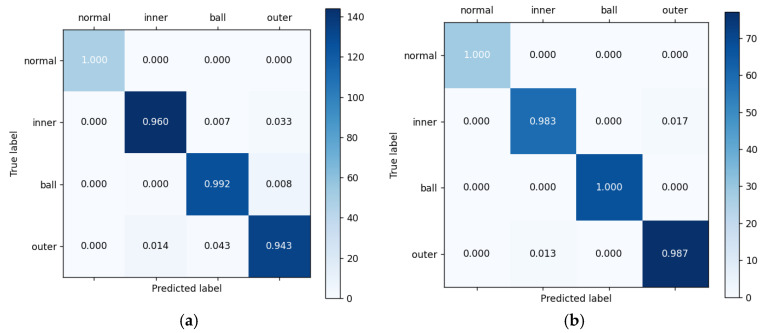
Confusion matrix for classification accuracy. (**a**) Real samples. (**b**) Generated samples.

The confusion matrices for model classification accuracy are presented in Figure 13. The classifier’s accuracy for real and test samples is 97.38% and 99.25%, respectively, indicating a high level of precision. This high accuracy allows the classifier to effectively guide the generator in producing samples of specific fault types, enhancing the overall performance and reliability of the TACGAN in fault diagnosis tasks.

**Figure 14:**
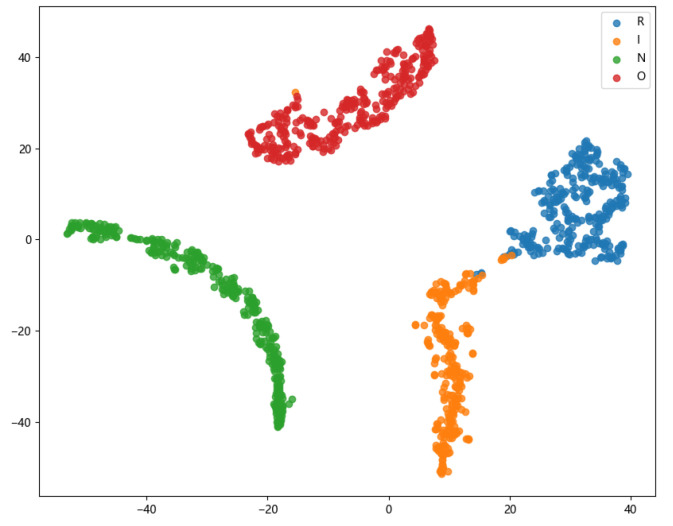
Feature visualization results for TACGAN.

Furthermore, to assess the feature-level performance, 2D feature visualization using t-SNE was conducted. The presented results in Figure 14 indicate that the samples generated by the TACGAN are well clustered in 2D space, with balanced representation across the four fault types. The clear separation of the four fault types by the TACGAN classifier further underscores the effectiveness and superiority of the TACGAN in generating high-quality samples.

#### 4.2.2. Comparison and Analysis of Sample Generation Effect

To validate the rationality and superiority of the TACGAN, it is compared with the ACGAN and WGAN-GP [42]. The ACGAN employs CNNs and leverages tagged samples, while the WGAN-GP is a standard GAN model utilizing the Wasserstein distance, incorporating both a discriminator and a generator. The maximum mean difference (MMD) metric is used to explore the similarity between the generated samples and the actual samples. The MMD works by mapping the distributions of the generated and real samples into another space, calculating the distances between each corresponding point, and summing these distances to measure the overall similarity between the two distributions.

(19) $$MMD(X,Y)={\left\|\overset{n_{1}}{\underset{i=1}{\sum }}\varphi \left(x_{i}\right)-\overset{n_{2}}{\underset{j=1}{\sum }}\varphi \left(y_{i}\right)\right\|}_{H}^{2}=\frac{1}{m\left(m-1\right)}\sum_{i\neq j}^{m}k\left(x_{i},x_{j}\right)+\frac{1}{n\left(n-1\right)}\sum_{i\neq j}^{n}k\left(y_{i},y_{j}\right)-\frac{2}{mn}\sum_{i,j=1}^{m,n}k\left(x_{i},x_{j}\right)$$

**Figure 15:**
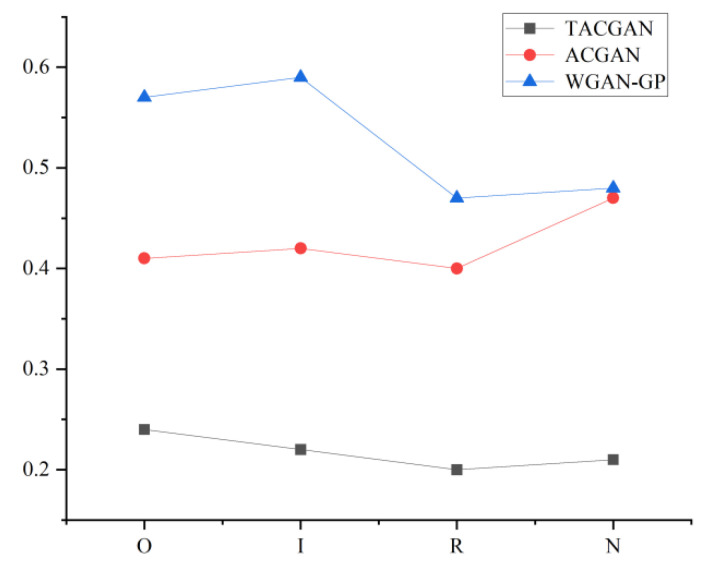
MMD distance of the data with 100 km/h.

In this study, Φ(·) denotes a high-dimensional mapping of vectors. Equation (19) indicates that a smaller MMD reflects a closer alignment between the spatial distributions of generated samples and real samples. For each bearing fault type, five real samples were randomly selected and used for comparison across three models: TACGAN, ACGAN, and WGAN-GP. This process generated 12 MMD distances, which are illustrated in Figure 15. The results show that the TACGAN consistently achieves the smallest MMD values (0.2403, 0.2219, 0.1982, 0.2115) across various fault types, indicating that the generated samples are more similar to real samples compared to those produced by the ACGAN and WGAN-GP.

**Figure 16:**
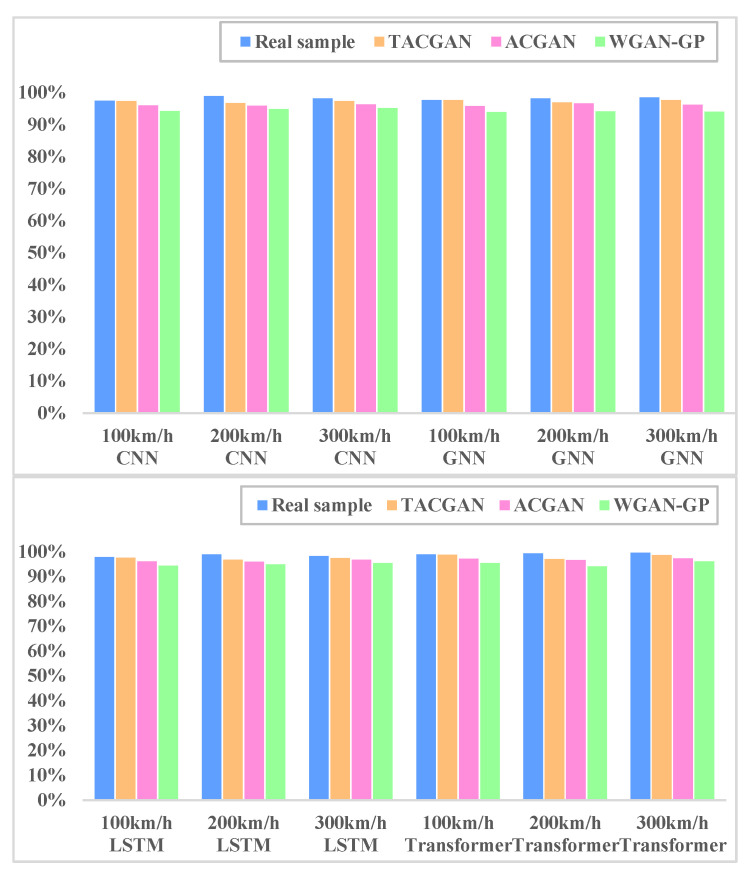
Accuracy of the different datasets.

To validate the accuracy of the datasets generated by the TACGAN, two fault diagnosis models, including Convolutional Neural Networks (CNNs), Graph Convolutional Networks (GNNs), Long Short-Term Memory Networks (LSTMs) and Transformer Networks [47,48,49,50,51,52], were employed. Both models were initially trained on sufficient real samples, and their diagnostic accuracy was recorded. As depicted in Table 5 and Figure 16, the diagnostic accuracy of the models trained on TACGAN-augmented datasets is comparable to that achieved with real datasets.

## 5. Conclusions

This study introduces a novel model for augmenting wheelset bearing fault samples, incorporating a transformer-based generator and discriminator, along with an independent fault classifier. The proposed TACGAN model exhibits several distinct advantages as follows:

(1) By employing a transformer network, the TACGAN bypasses the need for complex recursive structures. This approach allows for the direct extraction of both global and local features from input feature maps, thereby streamlining the model architecture and boosting computational efficiency.
(2) The TACGAN effectively learns and replicates the distribution of real samples within a high-dimensional space. This results in generated samples that closely mirror the properties of actual data, which is particularly advantageous when addressing diverse and intricate fault types.
(3) Testing has demonstrated that the TACGAN achieves an impressive 99% accuracy in augmenting wheelset bearing data. The fault samples produced by the TACGAN significantly enhance the dataset, improving the overall robustness and reliability of fault diagnosis systems.

## Figures and Tables

**Table 1:** Details of the parameters for the generator and discriminator.

Parameters	Value
Encoder Stacking Depth, N	5
Number of MSA Heads, h	4
Hidden Dimension of MLP, dMLP	256
Embedding Dimension, d	64
Batch Size	32
Token Sequence Length, G	65
Token Sequence Length, D	66

**Table 2:** Details of the parameters.

Networks	Layers	Parameter Setting	Operation/Activation
Classifier	Conv2D	3 × 3 × 32	BN + pooling/ReLu
	Conv2D	3 × 3 × 64	BN + pooling/ReLu
	Conv2D	3 × 3 × 128	BN + pooling/ReLu
	Conv2D	3 × 3 × 256	BN + pooling/ReLu
	Dense	256	None/ReLu
	Dense	128	None/ReLu
	Dense	Class number	None/Softmax

**Table 3:** Details of the test wheelset bearings.

Model Number	Pitch Diameter D/mm	Roller Diameter D/mm	Contact Angle φ/°	Number of Rolling Elements
197,726	176.29	24.76	8.83	20

**Table 4:** Details of datasets.

Fault Type	Speed Condition/km/h	Sample Size
N	100/200/300	2000/2000/2000
I	100/200/300	2000/2000/2000
O	100/200/300	2000/2000/2000
R	100/200/300	2000/2000/2000

**Table 5:** Diagnosis accuracy of each model in 100 km/h.

Model	Accuracy (Real Samples)	Accuracy (TACGAN)	Accuracy (ACGAN)	Accuracy (WGAN-GP)
CNN	97.46%	97.33	97.06	97.25
GNN	97.72%	97.68%	95.84%	93.93%
LSTM	97.82%	97.56%	96.12%	94.38%
Transformer	98.86%	98.83%	97.13%	95.47%

## Data Availability

The datasets presented in this article are not readily available because the data are part of an ongoing study.
